# Efficacy of Pirfenidone According to Dose in Patients with Idiopathic Pulmonary Fibrosis: A Prospective, Observational, Single-Center Cohort Study

**DOI:** 10.3390/life13112118

**Published:** 2023-10-26

**Authors:** Ho Young Lee, So Young Jung, Ji Hoon Jang, Junghae Ko, Dae-Wook Kim, Minyoung Her, Jae Ha Lee

**Affiliations:** 1 Division of Pulmonology, Department of Internal Medicine, Inje University Busan Paik Hospital, Inje University College of Medicine, Busan 47392, Republic of Korea; hoyoung@paik.ac.kr; 2 Division of Dermatology, Inje University Haeundae Paik Hospital, Inje University College of Medicine, Busan 48108, Republic of Korea; docjsy@hanmail.net; 3 Division of Pulmonology and Critical Care Medicine, Department of Internal Medicine, Inje University Haeundae Paik Hospital, Inje University College of Medicine, Busan 48108, Republic of Korea; saturn80396@gmail.com; 4 Division of Endocrinology, Department of Internal Medicine, Inje University Haeundae Paik Hospital, Inje University College of Medicine, Busan 48108, Republic of Korea; arrioph1@gmail.com; 5 Department of Orthopedic Surgery, Inje University Haeundae Paik Hospital, Inje University College of Medicine, Busan 48108, Republic of Korea; wook912@naver.com; 6 Division of Rheumatology, Department of Internal Medicine, Inje University Haeundae Paik Hospital, Inje University College of Medicine, Busan 48108, Republic of Korea; h00638@paik.ac.kr

**Keywords:** idiopathic pulmonary fibrosis, pirfenidone, disease progression

## Abstract

Background: Idiopathic pulmonary fibrosis (IPF) is a progressive fibrotic lung disease with a poor prognosis. Pirfenidone is approved and widely used for the treatment of IPF and reduces lung function decline. The aim of this study was to evaluate the efficacy of different doses of pirfenidone for the prevention of disease progression in patients with IPF. Methods: This was a prospective, observational, single-center cohort study conducted in Haeundae Paik Hospital, Republic of Korea, from April 2021 to March 2023. IPF patients were assigned to three groups according to the dose of pirfenidone (600 mg, 1200 mg, 1800 mg). Disease progression was defined as an absolute decline to ≥5% of forced vital capacity (FVC) (% predicted value) or an absolute decline to ≥10% of diffusing capacity of the lung for carbon monoxide (DLco) (% predicted value) over 12 months. The primary endpoint was to evaluate the clinical effects of pirfenidone of each dosage on disease progression in IPF patients by comparing the FVC (% predicted value) and DLco (% predicted value) values over 12 months. The secondary endpoint was to evaluate the prognostic value of Krebs von den Lungen-6 (KL-6) in the disease progression in IPF patients using the baseline KL-6 value and the change in KL-6 values between the baseline and 12 months. Results: A total of 44 patients were enrolled, of whom 39 completed the study, with 13 patients assigned to each of the three groups. The median age was 71.7 years, and 79.5% of patients were men. The baseline characteristics were similar across groups, except the 600 mg group was older (75.9 vs. 69.2 vs. 68.2 years, *p* = 0.016). The overall median change in FVC and DLco over 12 months was −2.7% (IQR: −9.1%, −1.2%) and −3.8% (IQR: −13.6%, −3.7%), respectively. There was no difference in the decline in FVC (change in FVC, % predicted value: −3.23 vs. −4.08 vs. −1.54, *p* = 0.621) and DLco (change in DLco, % predicted value: 0.00 vs. −3.62 vs. −3.15, *p* = 0.437) among the three groups. Fourteen patients (35.9%) suffered disease progression. The rate of disease progression did not differ according to the dose of pirfenidone (38.5 vs. 38.5 vs. 30.8%, *p* = 1.000). In multivariable logistic regression analysis, KL-6 was not a statistically significant predictor of disease progression. Conclusions: In our study, regardless of dose, consistent pirfenidone use for 12 months resulted in similar efficacy for the prevention of disease progression in patients with IPF. Large-scale, randomized, double-blind, placebo-controlled clinical trials are needed.

## 1. Introduction

Idiopathic pulmonary fibrosis (IPF) is a chronic, progressive, fibrosing interstitial pneumonia characterized by progression, which results in poor prognosis and high mortality [1,2]. The median survival following diagnosis in patients with IPF was 2–5 years without treatment [3,4]. In 2014, the first disease-modifying antifibrotic agents, including pirfenidone, were introduced and widely applied for the treatment of IPF [5,6]. Pirfenidone is an antifibrotic drug known to act on multiple fibrogenic pathways to reduce fibrosis in IPF patients with anti-inflammatory effects [6,7]. There is a growing body of evidence suggesting that pirfenidone may reduce lung-function decline and decline in distance during a six-minute walk test, and prolong disease progression-free survival [5,8,9]. Regarding pirfenidone dose, the maximal recommended dose of pirfenidone was differently approved in Korea/Japan (1800 mg/day) and Europe/the United States (2400 mg/day). In addition, maintaining the maximal dose of pirfenidone is difficult due to related adverse effects, and the ratio of maintaining the maximal dose of pirfenidone was less than 50% in the real-world practice [10]. Therefore, the efficacy of different doses of pirfenidone remains unclear, making it a highly significant topic. Only a few retrospective observational studies have been reported [11,12,13].

The mortality rate is an important primary outcome in the research of IPF; however, due to the limitation of infrequency during a limited period of a prospective study, other outcomes have been used as surrogate markers for predicting prognosis [14]. Among several risk factors for poor prognosis reported in previous studies [15,16,17,18], lung function decline is one of the most important risk factors [19,20]. However, diagnosis of disease progression by measuring lung function has the limitation of being confirmed after lung function has already deteriorated. Therefore, there has been increasing interest in and evidence of biomarkers being used to predict the prognosis of IPF early, as serum biomarkers can be relatively easily obtained at the diagnosis and during the follow-up period requiring minimal effort from patients. In addition, they can be measured repeatedly and less invasively than pulmonary function [21,22]. Krebs von den Lugen-6 (KL-6) is a mucin-like glycoprotein, produced by type 2 alveolar epithelial cells, that is one of the most widely used biomarkers in IPF [23,24]. Recently, Huang et al. reported that a higher level of KL-6 at baseline was significantly related to high mortality in 47 IPF patients receiving nintedanib (HR 5.39, 95% CI: 1.16–24.97, *p* = 0.031), and demonstrated a trend predicting faster lung function decline [25]. Therefore, in this study, we aimed to estimate the clinical efficacy of pirfenidone, according to three doses, in preventing disease progression, and the roles of baseline and change in KL-6 for predicting disease progression in IPF patients receiving pirfenidone. 

## 2. Materials and Methods

### 2.1. Study Design

This was a prospective, single-center, observational study conducted at Haeundae Paik Hospital, Republic of Korea, with IPF patients from April 2021 to March 2023. Patients were divided into three groups based on their daily dose of pirfenidone, which had been maintained for a minimum of six months before enrollment: (1) 600 mg, (2) 1200 mg, and (3) 1800 mg. All patients were informed of the results of the tests, including the KL-6 test performed every three months and the pulmonary function test during the sixth month of the study, and the patients in the 600 mg and 1200 mg groups had an opportunity to escalate their dose of pirfenidone. The duration of the study was 12 months. 

### 2.2. Study Subjects

The inclusion criteria were as follows: (1) Patients who were diagnosed with IPF according to the diagnostic criteria of the American Thoracic Society (ATS)/European Respiratory Society (ERS)/Japanese Respiratory Society/Latin American Thoracic Association statement [26]; (2) patients within five years of diagnosis of IPF; (3) patients who had maintained the fixed dosage of pirfenidone for at least six months and consented to continue the same dosage during the study period; and (4) patients who consented to participation in the study. The exclusion criteria were as follows: (1) Patients who refused to provide informed consent for the study; (2) patients who could not maintain the fixed dose of pirfenidone due to a drug-adverse event (including patients for whom pulmonary function decreased and were given the option to increase their dose of pirfenidone, and those for whom the duration of medication was less than six months); and (3) patients who did not undergo the necessary tests for the study. 

The study was conducted in accordance with the tenets of the Declaration of Helsinki. The study protocol was approved by the Institutional Review Board of Haeundae Paik Hospital (IRB number: 2021-05-049). Informed consent was obtained from all subjects prior to enrollment.

### 2.3. Clinical Data

Clinical data for all enrolled patients were prospectively collected. Spirometry, diffusing capacity for carbon monoxide (DLco), and the six-minute walk test (6MWT) were performed every six months from enrollment. Spirometry and DLco were measured according to the ATS/ERS recommendation, and the results were presented as a percentage of the normal predicted value [27,28,29]. The 6MWT was conducted in accordance with previously published guidelines [30]. Laboratory tests, including KL-6, were measured every three months using the Nanopia KL-6 assay (SEKISUI MEDICAL, Tokyo, Japan). Disease progression was defined as an absolute decline to ≥5% of forced vital capacity (FVC) (% predicted value) or an absolute decline to ≥10% of DLco (% predicted value) over 12 months [19]. All pirfenidone-related adverse events and IPF-related adverse events during the study period were recorded.

### 2.4. Endpoints

The primary endpoint was to assess the clinical effects of pirfenidone according to each dosage (600 mg, 1200 mg, and 1800 mg) on the disease progression in patients with IPF by comparing the baseline, 6-month, and 12-month % predicted values of FVC and DLco. The secondary endpoint was to evaluate the prognostic value of KL-6 in the disease progression in IPF patients. We investigated whether the baseline KL-6 value and the change in KL-6 values between baseline and 12 months could predict the disease progression.

### 2.5. Statistical Analysis

Variables were summarized as frequency and percentage for categorical data, and mean ± standard deviation and median (interquartile range (IQR)) for numeric data. Group differences were tested using the chi-square test or Fisher’s exact test for categorical data, and independent t-test, analysis of variance, Mann–Whitney U test, or Kruskal–Wallis test for numeric data as appropriate. To determine whether distributions were normal, we used the Shapiro–Wilk test. We used error bar charts, spaghetti plots, and line graphs to visualize data. Univariate and multivariate logistic regression analyses were performed to identify prognostic factors independently related to disease progression. Considering the nature of the repeated measured data, a generalized linear mixed model (GLMM) with random intercepts was used. The GLMM model included repeated measures of numeric variables as dependent variables: group, time, and group x time interaction as fixed effects and subject as a random effect. To avoid assumptions about the covariance structure, we used an unstructured covariance matrix that was allowed to differ across groups for the GLMM analysis. All statistical analyses were carried out using SPSS 26.0 statistical software (IBM Corp., Armonk, NY, USA) and R statistical software (version 3.4.0; R Foundation, Vienna, Austria, http://www.r-project.org/ (accessed on 1 June 2023). *p*-values less than 0.05 were considered statistically significant.

## 3. Results

### 3.1. Study Population and Baseline Characteristics

**Figure 1:**
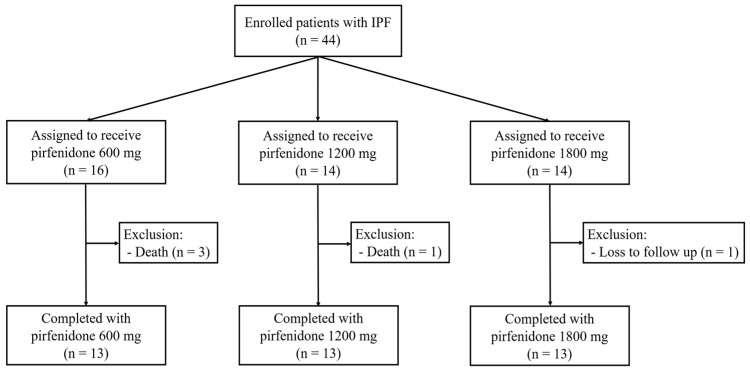
Flowchart of patient inclusion, allocation based on the dosage of pirfenidone, and the distribution of completed patients. IPF, idiopathic pulmonary fibrosis.

We enrolled a total of 44 patients, and overall, five patients were excluded due to death (four patients) and drop-out (one patient) (Figure 1). A total of 12 patients (27.3%) were diagnosed with IPF via pathologic exam (surgical lung biopsy and transbronchial lung cryobiopsy). Finally, 13 patients were assigned to each group, and 39 patients completed the study.

The median patient age was 71.7 years (IQR: 66.0–75.0 years), and 79.5% of patients were male. Most patients (82.1%) were ever-smokers. Most patients showed mild restrictive ventilatory defects (median: 72.0% predicted, range 66.0–83.0%) and reduced DLco (median: 59% predicted, range: 51.0–83.0%). The 600 mg group was significantly older than the other groups (median age: 75.9 vs. 69.2 vs. 68.2 years, *p* = 0.016). The other baseline characteristics were similar among the three groups (Table 1). Three patients died during the study period (600 mg group: 3 patients, 1200 mg group: 1 patient), with all three among the group of six patients who suffered acute exacerbation (AE). There were no differences in rates of pirfenidone-related adverse events among the three groups (rate %: 61.5% vs. 30.8% vs. 23.1%, *p* = 0.103). The most common pirfenidone-related adverse event was anorexia (93.3%), followed by photosensitivity (26.7%), and itching (20.0%).

### 3.2. Primary Endpoint

**Figure 2:**
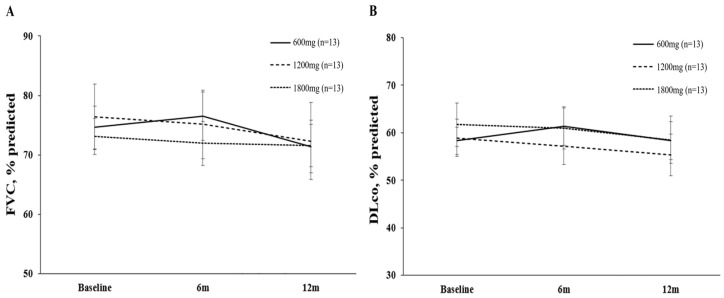
Changes in FVC and DLco for each group over a 12-month period. (**A**) Changes in FVC % predicted over 12 months for each pirfenidone group. (**B**) Changes in DLco % predicted over 12 months for each pirfenidone group. FVC, forced vital capacity; DLco, diffusing capacity of the lung for carbon monoxide.

The overall median change in FVC over 12 months was −2.7% (IQR: −9.1%, −1.2%). There was no difference in FVC decline among the three groups (change in FVC, % predicted value: −3.23 vs. −4.08 vs. −1.54, *p* = 0.621) (Table 2, Figure 2). The overall median change in DLco over 12 months was −3.8% (IQR: −13.6%, −3.7%). There were no differences in DLco decline among the three groups (change in DLco, % predicted value: 0.00 vs. −3.62 vs. −3.15, *p* = 0.437).

**Figure 3:**
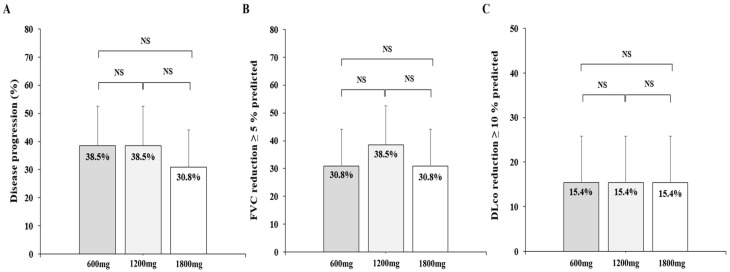
Rate of disease progression, FVC decline ≥5%, and DLco decline ≥10% for each pirfenidone group. (**A**) The proportion of patients with disease progression for each pirfenidone dosage group, (**B**) The proportion of patients with FVC decline ≥5% predicted for each pirfenidone dosage group, (**C**) The proportion of patients with DLco decline ≥10% predicted for each pirfenidone dosage group. NS, no statistical significance; FVC, forced vital capacity; DLco, diffusing capacity of the lung for carbon monoxide.

A total of 14 patients (35.9%) suffered disease progression (Figure 3). There were no differences in the rate of disease progression among the three groups (38.5% vs. 38.5% vs. 30.8%, *p* = 1.000). In multivariate logistic regression for predicting disease progression, female gender was a statically significant risk factor (odds ratio (OR) 0.09, 95% confidence interval (CI): 0.01–0.063, *p* = 0.015) (Table 3).

### 3.3. Secondary Endpoint

The median value of KL-6 in all patients was 668.1 U/mL (IQR: 465.3–924.5 U/mL). The baseline value and changes in KL-6 between baseline and 12 months were similar among the three groups (median: 496.6 vs. 735.0 vs. 698.1 U/mL, *p* = 0.174). However, in the logistic regression analysis, both baseline KL-6 value and changes in KL-6 between baseline and 12 months were not statistically significant risk factors for predicting disease progression. In a sub-group analysis of the baseline values of 27 patients (77.1%) with KL-6 ≥ 500 U/mL, the relative change in KL-6 showed a tendency to predict disease progression in multivariate logistic regression analysis (OR 1.03, 95% CI: 1.00–1.06, *p* = 0.74) (Table 4). 

## 4. Discussion

This study was the first prospective study to demonstrate the efficacy of pirfenidone according to dose in disease progression, defined as the decline of FVC to ≥5% or DLco to ≥10% over 12 months in patients with IPF. We observed no significant differences in the disease progression rate according to the dose of pirfenidone over 12 months. Female gender was the only risk factor for disease progression in multivariate logistic regression analysis. KL-6 did not predict disease progression.

For East Asian patients with IPF, pirfenidone was approved with a recommended maximum dose of 1800 mg per day based on a previous study in Japan, in contrast to the recommended maximum dose in Europe and the United States, which is 2400 mg [6,31,32]. However, previous post-marketing data reflecting real-world clinical settings showed that many East Asian patients with IPF are unable to take the recommended maximum dose of pirfenidone due to related adverse events [13,33]. To address this issue, previous research sought to estimate the efficacy of lower doses of pirfenidone for preventing lung function decline [11,12]. Lee et al., in a retrospective, single-center study including 295 patients with IPF, showed that there was no difference in change in FVC (mean change, % predicted: −2.12 vs. −1.24, *p* = 0.730) and DLco (mean change, % predicted: −2.92 vs. 2.71, *p* = 0.490) between the full-dose group (1800 mg of pirfenidone) and the low-dose group (600 mg or 1200 mg of pirfenidone) [12]. Song et al., in a retrospective study including 234 patients with IPF, presented that both groups of low-dose pirfenidone (<1200 mg/day) and high-dose pirfenidone (≥ 1200 mg/day) were useful in preventing FVC decline compared to the control group (no pirfenidone use); however, there was no difference between the low-dose and high-dose groups (*p* = 0.976) [13]. However, these studies have limitations such as retrospective design, small samples of research to review, and possible biases such as dose adjustments and different durations of fixed doses [11,12,13,33]. Therefore, we conducted a prospective study of IPF patients who maintained the same durations of fixed-dose administration of pirfenidone and the same evaluation period to determine lung function decline. 

The most well-supported effect of pirfenidone is the reduction in lung function decline, which has been demonstrated through a number of previous studies [5,6,34]. Our results showed that, regardless of dose, pirfenidone prevented lung function decline over 12 months, agreeing with the results of previous studies. A previous multicenter retrospective study of 338 non-standard patients with IPF who had received less than 1800 mg of pirfenidone showed a consistent effect on the prevention of disease progression (mean difference of FVC, % predicted and DLco, % predicted per year before and after treatment: 2.13 vs. 3.17%, *p* = 0.307) compared to a standard group who had received 1800 mg of pirfenidone [11]. However, this result does not imply that maintaining the recommended maximum dose of pirfenidone is not imperative, but rather emphasizes the importance of the best efforts to maintain the highest feasible dose of pirfenidone continuously, considering each patient’s characteristics, and pirfenidone-related adverse events in real practice. 

Disease progression is one of the most crucial prognostic factors of mortality, and recent international guidelines defined a decline in FVC greater than 5% or in DLco greater than 10% over 12 months as one criterion of lung function decline [19]. In this study, the baseline values and changes in KL-6 were not significant predictive factors for disease progression except in sub-group analysis of patients with KL-6 values over 500 U/mL, among whom change in KL-6 between baseline and 12 months showed a tendency for predicting disease progression. In our study, the baseline KL-6 value was lower than 500 U/mL in 30.8% of patients. Previous studies reported conflicting results compared to our study, regarding KL-6 as one of the best biomarkers for predicting disease progression [35,36,37]. Our results may conflict with the results of previous studies showing the predictive capacity for disease progression in patients with IPF due to the substantial number of patients with baseline values less than 500 U/mL of KL-6 in this study, as well as the small total number of enrolled patients. Previous studies suggested a diagnostic cut-off value of IPF around 500 U/mL, and the predictive capacity for disease progression might be greater for higher values of KL-6 [36,38,39]. One previous study showed similar results to our study [35]. In a retrospective study of 205 patients with fibrotic ILDs (49% IPF) showing the effectiveness of KL-6 (Hazard ratio [HR] 5.687, 95% CI: 1.000–1.000, *p* = 0.017) in lowering the risk of disease progression in a Cox regression analysis, a higher level of KL-6 > 1300 U/mL was associated with the prediction of disease progression. In addition, the different definitions of disease progression we used compared to previous studies might contribute to these conflicting results [35,36,37]. Additional research is needed to further assess the role of KL-6 in disease progression. 

Generally, it is known that the male gender of patients with ILD is associated with a poor prognosis, such as a higher mortality rate and higher risk of disease progression [40]. This is well demonstrated in the GAP model, which has been used widely to predict the prognosis in patients with IPF [41]. Regarding disease progression, the male gender is at increased risk of disease progression [42,43]. However, it is not well known whether the differences in prognosis between genders are mainly impacted by behavior or biological factors of gender. In a Canadian registry of patients with fibrotic ILD, the male gender was associated with a shorter time to treatment initiation than the female gender [44]. In addition, in a US administrative claims-based cohort of patients with IPF, males had a higher rate of antifibrotic treatment than women (30% vs. 21.9%, *p* < 0.001) [45]. In contrast to previous research, this study found that female gender was a risk factor for disease progression. This conflicting result might be assumed to be a result of the absence of female patients in the 1800 mg group and the limitation of a small number of patients. 

There are some limitations to this study. First, it was a single-center cohort study with a small number of patients, which limited the generalizability of our findings. However, the baseline characteristics and results were similar overall to those of patients in other studies [11,12,13,25]. Second, we defined disease progression solely as lung function decline, excluding criteria such as symptoms and radiologic evidence. However, in this study, the rate of disease progression was 35.9%, which was not lower than previous studies [6,46]. Therefore, this difference should have minimal impact on the primary endpoint of lung function decline. Third, due to the prospective nature of this study aimed at prohibiting dose changes of pirfenidone, there is possible bias because IPF patients with relatively stable and low rates of pirfenidone-related adverse events were included. Fourth, due to the limitation of the short duration of this prospective study (12 months), this study could not demonstrate the impact of pirfenidone according to different doses on mortality and acute exacerbation. Further study is needed to prove the impact on prognosis, such as mortality and prevention of exacerbation in patients with IPF. 

## 5. Conclusions

We demonstrated no significant differences in disease progression rate among IPF patients who were assigned fixed doses of 600 mg, 1200 mg, or 1800 mg of pirfenidone over 12 months. This result emphasizes the importance of consistently maintaining the maximum feasible dose of pirfenidone in real practice. Further large-scale research is needed to elucidate the role of KL-6 in predicting disease progression.

## Figures and Tables

**Table 1:** Comparison of baseline characteristics according to pirfenidone dosage.

Variable	Overall		Group		*p*-Value
		600 mg	1200 mg	1800 mg	
Patient, no.	39 (100.0)	13 (33.3)	13 (33.3)	13 (33.3)	
Age, years	69.0 (66.0–75.0)	75.0 (68.0–83.5)	68.0 (65.5–71.5)	68.0 (66.5–70.0)	0.016
Male	31 (79.5)	10 (76.9)	8 (61.5)	13 (100.0)	0.065
Ever-smokers	32 (82.1)	10 (76.9)	9 (69.2)	13 (100.0)	0.128
BMI, kg/m^2^	24.7 (23.1–26.4)	24.9 (23.1–26.7)	24.7 (22.6–26.8)	24.7 (23.6–26.7)	0.812
Pulmonary function					
FVC, % predicted	72.0 (66.0–83.0)	73.0 (66.5–79.5)	71.0 (60.5–88.0)	72.0 (63.0–84.0)	0.857
DLco, % predicted	57.0 (51.0–70.0)	54.0 (52.5–66.5)	53.0 (48.5–73.0)	62.0 (44.5–75.5)	0.807
FEV1/FVC	79.0 (74.0–83.0)	76.0 (71.5–83.0)	80.0 (69.5–88.0)	80.0 (77.0–83.5)	0.378
Six-minute walk test					
Distance, m	498.0 (444.0–548.3)	465.0 (430.0–515.0)	500.0 (388.5–552.0)	514.0 (462.8–561.0)	0.282
Baseline SpO_2_, %	96.0 (95.0–97.0)	96.0 (95.0–97.0)	96.0 (96.0–97.0)	95.5 (94.0–97.8)	0.651
Lowest SpO_2_, %	91.5 (87.0–94.8)	93.0 (87.0–95.0)	91.0 (88.0–94.0)	91.0 (85.5–94.3)	0.473
KL-6, U/mL	665.8 (481.3–874.1)	497.7 (306.9–782.0)	714.6 (531.2–1045.8)	703.6 (497.4–888.0)	0.174
Drug adverse events	15 (38.5)	8 (61.5)	4 (30.8)	3 (23.1)	0.103
AE	3 (7.7)	0(0.0)	0 (0.0)	3 (23.1)	0.094
Death	4 (9.1)	3 (23.1)	1 (7.7)	0 (0.0)	0.104

Data are presented as median (IQR) or number (%) unless otherwise indicated. BMI, body mass index; FVC, forced vital capacity; DLco, diffusing capacity of the lung for carbon monoxide; FEV1, forced expiratory volume in one second; KL-6, Krebs von den Lungen-6; AE, acute exacerbation.

**Table 2:** Comparison of changes in the FVC and DLco in the three groups according to pirfenidone dosage.

Variable		Group		*p*-Value
	600 mg (*n* = 13)	1200 mg (*n* = 13)	1800 mg (*n* = 13)	
FVC, % predicted				
baseline	73.0 (66.5–79.5)	71.0 (60.5–88.0)	72.0 (63.0–84.0)	0.857
6 months	74.0 (69.0–82.5)	71.0 (60.0–90.0)	73.0 (60.5–85.0)	0.768
12 months	71.0 (59.0–81.5)	64.0 (57.0–87.0)	74.0 (60.0–83.0)	0.990
DLco, % predicted				
baseline	54.0 (52.5–66.5)	53.0 (48.5–73.0)	62.0 (44.5–75.5)	0.807
6 months	61.0 (51.0–78.0)	52.0 (46.5–70.5)	63.0 (46.5–70.0)	0.734
12 months	56.0 (47.0–72.0)	59.0 (41.5–65.5)	58.0 (39.0–74.0)	0.851

Values are presented as median (IQR). FVC, forced vital capacity; DLco, diffusing capacity of the lung for carbon monoxide.

**Table 3:** Logistic regression analysis for prognostic factors for disease progression in patients with IPF (Total patients).

Variable	Unadjusted Analysis		Multivariable Analysis	
	OR (95% CI)	*p*-Value	OR (95% CI)	*p*-Value
Age	0.97 (0.88–1.06)	0.472		
Male	0.12 (0.02–0.70)	0.018	0.12 (0.02–0.70)	0.018
Smoking	0.34 (0.06–1.82)	0.207		
BMI	0.78 (0.60–1.02)	0.065		
Pulmonary function				
FVC, % predicted	0.98 (0.93–1.02)	0.326		
DLco, % predicted	0.98 (0.93–1.03)	0.478		
Six-minute walk test				
Distance	0.99 (0.99–1.00)	0.189		
Lowest SpO_2_	0.95 (0.83–1.09)	0.453		
Baseline KL-6	1.00 (1.00–1.00)	0.496		
Δ KL-6, % *	1.03 (1.00–1.06)	0.077		

* The relative change in KL-6 value from baseline to 12 months. IPF, idiopathic pulmonary fibrosis; OR, odds ratio; CI, confidence interval; BMI, body mass index; FVC, forced vital capacity; DLco, diffusing capacity of the lung for carbon monoxide; SpO_2_, percutaneous oxygen saturation; KL-6, Krebs von den Lungen-6.

**Table 4:** Logistic regression analysis for prognostic factors for disease progression in IPF patients with high baseline KL-6 levels (≥500 U/mL).

Variable	Unadjusted Analysis		Multivariable Analysis	
	OR (95% CI)	*p*-Value	OR (95% CI)	*p*-Value
Age	0.97 (0.84–1.11)	0.644		
Male	0.17 (0.03–1.14)	0.069	0.09 (0.01–0.63)	0.015
Smoking	0.38 (0.05–2.78)	0.342		
BMI	0.59 (0.36–0.96)	0.034		
Pulmonary function				
FVC, % predicted	0.98 (0.92–1.03)	0.434		
DLco, % predicted	0.98 (0.92–1.04)	0.573		
Six-minute walk test				
Distance	1.00 (0.99–1.01)	0.548		
Lowest SpO_2_	0.97 (0.83–1.12)	0.645		
Baseline KL-6	1.00 (1.00–1.00)	0.749		
Δ KL-6, % *	1.04 (1.00–1.08)	0.043	1.03 (1.00–1.06)	0.074

* The relative change in KL-6 value from baseline to 12 months. IPF, idiopathic pulmonary fibrosis; KL-6, Krebs von den Lungen-6; OR, odds ratio; CI, confidence interval; BMI, body mass index; FVC, forced vital capacity; DLco, diffusing capacity of the lung for carbon monoxide; SpO_2_, percutaneous oxygen saturation.

## Data Availability

All data are contained within the manuscript.
